# Outpatient balloon catheter vs inpatient prostaglandin for induction of labour (OBLIGE): a randomised controlled trial

**DOI:** 10.1186/s13063-020-4061-5

**Published:** 2020-02-17

**Authors:** Michelle R. Wise, Joy Marriott, Malcolm Battin, John M. D. Thompson, Michael Stitely, Lynn Sadler

**Affiliations:** 1 Department of Obstetrics and Gynaecology, University of Auckland, PO Box 92019, Auckland, 1142 New Zealand; 2 Newborn Services, Auckland District Health Board and Department of Paediatrics, University of Auckland, Auckland, New Zealand; 3 Department of Women’s and Children’s Health, Dunedin School of Medicine, University of Otago, Dunedin, New Zealand; 4 Women’s Health, Auckland District Health Board, 2 Park Road, Grafton, Auckland, 1023 New Zealand

**Keywords:** Labour, induced, Cervical ripening, Balloon catheter, Prostaglandins, Randomised controlled trial

## Abstract

**Background:**

Approximately one in four pregnant women undergo an induction of labour. The purpose of this study is to investigate the clinical effectiveness, safety, and cost-effectiveness for mothers and babies of two methods of cervical ripening – inpatient care for women starting induction with vaginal prostaglandin E2 hormones, or allowing women to go home for 18 to 24 h after starting induction with a single-balloon catheter.

**Methods/design:**

This is a multi-centre randomised controlled trial in New Zealand. Eligible pregnant women, with a live singleton baby in a cephalic presentation who undergo a planned induction of labour at term, will be randomised to outpatient balloon-catheter induction or in-hospital prostaglandin induction. The primary outcome is caesarean section rate. To detect a 24% relative risk reduction in caesarean rate from a baseline of 24.8%, with 80% power and 5% type 1 error, will require 1552 participants in a one to one ratio.

**Discussion:**

If outpatient balloon-catheter induction reduces caesarean section rates, has additional clinical benefits, and is safe, cost-effective, and acceptable to women and clinicians, we anticipate change in induction of labour practice around the world. We think that home-based balloon-catheter induction will be welcomed as part of a patient-centred labour-induction care package for pregnant women.

**Trial registration:**

Australia New Zealand Clinical Trials Registry (ANZCTR), ACTRN: 12616000739415. Registered on 6 June 2016.

## Background

### Introduction

Induction of labour (IOL) is a common intervention in childbirth. Globally, approximately one in four pregnant women undergo an IOL, with significant variation by country [1]. IOL is defined as the artificial initiation of labour [2]; the alternative is expectant management of the pregnancy where spontaneous labour is awaited. IOL is offered when evidence shows that the benefit to mother and/or baby of an earlier planned birth outweighs the risks of induction. Common clinical scenarios for IOL include women with pre-labour rupture of membranes, or whose pregnancy is more than 1 week beyond their expected due date.

In New Zealand (NZ), the proportion of women who undergo an IOL has steadily increased, from 19.4% in 2006 to 24.0% in 2015 [3]. Compared to spontaneous onset of labour, IOL is associated with a prolonged length of hospital stay and with increased rates of epidural insertion, caesarean section, and post-partum haemorrhage. IOL has an impact on the woman’s experience of labour and birth, with women having to reconsider their birth plans and in some cases their planned place of birth. Women have identified that more pain relief during labour is required, they feel less positive about their birth experience and find the induction process challenging [4–6]. IOL also has an impact on hospital and staff resources, with a significant occupancy of acute beds and allocation of clinical resources for the obstetric unit. There is the potential for service commitments of IOL care to impact on the safe and efficient care to other women accessing acute maternity and gynaecological care.

In NZ, most women have their induction commenced with pharmacological methods. The most common method of cervical ripening (making the cervix ready for induction) involves one or more doses of prostaglandin (PG) E2 gel inserted into the vagina at regular intervals or a PGE2 controlled-release pessary that is inserted into the vagina and remains in situ for 12–24 h. This is followed by artificial rupture of membranes (breaking the waters) (ARM) and an intravenous infusion of oxytocin to stimulate uterine contractions. The vast majority of women will remain in hospital throughout. Prostaglandins (PGs) for cervical ripening are associated with the complication of uterine hyperstimulation (defined as contractions every minute or two or contractions lasting longer than 2 minutes, and associated with fetal distress; reported to occur about 5% of the time) [7].

### Literature review

There is moderate-quality evidence that mechanical methods (such as a balloon catheter) are a safe and effective alternative to pharmacological methods to ripen the cervix and induce labour. The Cochrane review on mechanical methods included 17 randomised controlled trials (RCTs) (1894 women) comparing IOL with mechanical methods to PGs [8]. Women undergoing IOL with a balloon catheter had less uterine hyperstimulation (0% vs 5%, risk ratio (RR) 0.16 (0.06–0.39)), and fewer instrumental vaginal births (21% vs 27%, RR 0.79 (0.64–0.98)). Balloon-catheter inductions were associated with more oxytocin usage (75% vs 50%). Both methods had comparable rates of vaginal birth not achieved within 24 h (RR 1.97 (0.43–8.95)), and of caesarean section (26% vs 23%, RR 1.07 (0.91–1.25)).

Diederen et al. performed a meta-analysis of 26 studies (8292 women) who had undergone cervical ripening with a balloon catheter [9]. The most common adverse event was participant pain/discomfort, which occurred in 3 in 1000 participants. This study suggests that the risk of adverse events, such as uterine hyperstimulation, during the period between insertion and expulsion of a balloon catheter in cervical ripening is very low (ranging from 0.0 to 0.26%). The authors concluded that further evaluation and implementation of this procedure in an outpatient setting for low-risk pregnancies was needed.

There is low-quality evidence that outpatient care during the cervical ripening phase of IOL is an alternative to inpatient care. The Cochrane review included four RCTs comparing outpatient to inpatient labour induction [10]. Three used PGs; one used a balloon catheter. Sciscione et al. randomised 111 women to outpatient vs inpatient balloon catheter and found no differences between the groups in their primary outcome (change in Bishop score, a clinical assessment of cervical ripening) or secondary outcomes [11]. There were no adverse events or maternal morbidity in this small study. Women in the outpatient group spent 10 fewer hours in hospital. An unanticipated finding was the suggestion that fewer women in the outpatient group needed a caesarean section (29% vs 43%, RR 0.67 (95% CI 0.41–1.10)). The authors suggested that this may be attributed to clinician and patient perception of a longer IOL process in the inpatient group, which might have led to an earlier diagnosis of ‘failed induction’ in the inpatient group. The authors of this Cochrane review called for more trials examining IOL in outpatient settings to assess its safety and effectiveness.

There may be benefits for women to undergo balloon-catheter induction as an outpatient. Henry et al. randomised 101 women in an Australian tertiary hospital to outpatient balloon catheter vs inpatient PGs to assess feasibility, clinical effectiveness and acceptability [12]. Women randomised to outpatient balloon catheter were less likely to achieve vaginal birth within 12 h of admission (28% vs 53%, *p* = 0.01); however, they had comparable rates of caesarean section (34% vs 29%, *p* = 0.6). They spent 11 fewer hours in hospital, had less discomfort (26% reported feeling a lot of discomfort vs 58%, *p* = 0.03) and more hours of sleep (5.8 vs 3.4, *p* < 0.01), felt more able to relax (*p* = 0.01) and to rest (*p* = 0.01), and were more likely to choose this method of IOL again (65% vs 42%, *p* = 0.03). Two other trials in Canada and Australia also show women’s preference for, and satisfaction with, outpatient management [13, 14].

The authors of many of the above studies concluded that a large study was needed to investigate the safety and effectiveness of outpatient balloon-catheter induction. We chose the comparator of inpatient induction with PGE2 because this is the most common method of cervical ripening in NZ (which would facilitate participation in the study). Moreover, due to the 5% risk of uterine hyperstimulation, we felt that it would be safer to keep women in hospital for monitoring throughout (also usual care in most hospitals in NZ).

A search of the ANZCTR identified one registered RCT answering the same research question, with a different primary outcome (ACTRN12614000039684). This trial is being led by Dr. M Beckmann in Australia, and the two principle investigators (PIs) have been in regular communication during the progress of both trials.

### Aim

To assess the clinical effectiveness, safety, and cost-effectiveness for mothers and babies of outpatient IOL commencing with a balloon catheter vs inpatient IOL with vaginal PGE2.

### Primary hypothesis

Our primary hypothesis is that women undergoing outpatient IOL with balloon catheter will have a lower caesarean section rate, compared to women having inpatient IOL with vaginal PGE2.

### Secondary hypotheses

Our secondary hypotheses are that outpatient balloon-catheter IOL will not result in increased adverse events for mother or baby; that outpatient balloon-catheter IOL will be associated with increased satisfaction; that outpatient balloon-catheter IOL will be associated with increased staff satisfaction; and that outpatient balloon-catheter IOL will be more cost-effective compared to inpatient IOL with vaginal PGE2.

## Methods/design

### Design

This is a multi-centre, randomised controlled superiority trial.

### Setting

Ten public hospitals around NZ are currently participating in the OBLIGE trial. These are Auckland, Tauranga, Whakatane, North Shore, Waitakere, Waikato, Wellington, Hawkes Bay, Taranaki, and Hutt Valley Hospitals. These hospitals serve urban, regional, and rural areas, and account for 50% of all women giving birth in NZ [3]. The number of women giving birth per year varies from 1514 in Taranaki to 7557 at North Shore/Waitakere [3].

### Characteristics of participants

The target population is pregnant women who develop one or more obstetric risks or complications where expedited birth by IOL is recommended by their clinician.

Women included in the study are pregnant women with a live singleton baby in a cephalic presentation; planning IOL at ≥ 37 weeks’ gestation, intact membranes, normal cardiotocography (CTG), Bishop score < 7, able to remain within 1 h of hospital and to have with them someone who can speak sufficient English to communicate with the hospital midwife on the telephone. A vaginal assessment of the cervix is required to establish the Bishop score, and a CTG also needs to be performed, in order to fully assess eligibility.

Women excluded from the study are women who have undergone previous caesarean; major fetal congenital anomaly; suspected fetal growth restriction; and maternal or fetal condition where the clinician feels that outpatient care would be contraindicated.

### Intervention and comparison

#### Outpatient balloon catheter

The hospital clinician will perform a speculum or vaginal examination (clinician preference) and place the single-balloon Foley catheter through the cervix to above the internal cervical os. The balloon will be inflated with 50 mL sterile saline and the catheter secured to the thigh with slight tension. The woman does not need a routine CTG after placement, unless there is clinical concern or to comply with local protocol. The woman will be asked to rate her discomfort with balloon-catheter placement on a numeric pain rating scale (NPRS) of 0 (no pain) to 10 (worst possible pain).

Before discharge from the unit, the woman will receive detailed verbal information about what to expect, to return to the hospital at any time if she has spontaneous rupture of membranes, contractions, bleeding or reduced fetal movements, and to contact the hospital midwives if concerned. The woman will also receive the OBLIGE written pamphlet on the outpatient balloon catheter with instructions to return to the unit at a specified time, 18–24 h after balloon-catheter placement.

From the time of return to hospital, the woman will remain an inpatient. The hospital clinician will perform a CTG and an ARM. The woman will be asked to rate her discomfort with ARM on a NPRS of 0 (no pain) to 10 (worst possible pain). If ARM is not possible, then the woman will receive PGs (alternative/second method of cervical ripening). Timing of ARM will occur according to clinical prioritisation and acuity in the unit, as for any women in the unit undergoing IOL.

#### Inpatient prostaglandins

The hospital clinician will place either a PGE2 gel (Prostin) or controlled-release pessary (Cervidil) in the vagina. Either method of administration is appropriate (hospital preference). The woman will be asked to rate her discomfort with PG placement on a NPRS of 0 (no pain) to 10 (worst possible pain). The woman does not need a routine CTG after placement, unless there is clinical concern, or to comply with local protocol. After 6 h (for gel) or 12–24 h (for pessary), the hospital midwife will perform a CTG, followed by an ARM. The woman will be asked to rate her discomfort with the ARM on a NPRS of 0 (no pain) to 10 (worst possible pain). If ARM is not feasible due to a low Bishop score, they will either place another PG or switch to second method. The Bishop score should be documented with each exam. Every PG should be prescribed on the National Medication Chart.

The assessment and administration of PG will be repeated regularly (not < 6 h for gel; not < 12 h for pessary) until ARM is possible, or the woman is established in labour or spontaneous rupture of membranes occurs, or the clinical team prefers to change to the second/alternative method, or to a maximum of six doses for gel or two doses for pessary. The timing and dose of each follow-up PG dose can be at the discretion of the clinical team. The date and time of the first and last PG will be documented. Whenever the woman starts to feel regular strong painful contractions, the midwife will perform a CTG. If ARM is still not possible, then the woman will be offered a balloon catheter (second method of cervical preparation) and remain an inpatient. However, if neither ARM nor balloon-catheter insertion is possible, then a senior clinician will decide how to proceed.

#### Discontinuing allocated intervention

Based on previous trials, we anticipate that some women will request removal of the catheter due to discomfort. Some women after randomisation may choose not to continue to participate in the trial for other reasons. If a participant withdraws, we will seek her consent to continue to have her data collected from her clinical notes.

#### Adherence to intervention protocols

Members of the research team at each hospital will be available to answer questions about the study protocols and reinforce the importance of adhering to the study protocols. Each hospital will embed reminders into their usual protocols and medical records.

#### Relevant concomitant care and interventions

Following ARM or spontaneous rupture of membranes, in the absence of spontaneous onset of strong regular contractions within 1–2 h, intravenous infusion of oxytocin will be started and titrated to the contractions, according to local hospital protocol. It should not be started < 6 h from insertion of the last PG gel or < 12 h from last PG pessary (if applicable). Continuous CTG monitoring is recommended whilst on oxytocin. Clinical care to participants will be provided by multiple clinicians throughout their induction, labour, birth, and post-partum. It is up to the discretion of the clinician if, during a cervix assessment, they also perform membrane sweeping. Established labour (defined for the purposes of this study as regular, strong, painful contractions and cervix dilated 4 cm or more) will be managed by the participant’s clinicians as per local protocol. Analgesia will be administered according to maternal request as per local practice. Additional care for study participants outside of routine labour and birth care is the collection of paired cord-blood samples. Each hospital has committed to not offer outpatient balloon-catheter IOL outside of this study for the duration of the study.

### Primary outcome

Caesarean section:

the proportion of participants who give birth by caesarean section.

### Secondary outcomes

*NOTE: all outcomes listed are as recommended for an IOL study by COSIOL* [15].

Maternal:

### Measured at time of induction of labour

For balloon catheter only:

- Duration in situ (time in hours will be calculated at the end of the study, based on times recorded by the staff member (from time of placement to time of removal/falling out)
- Balloon catheter removed or fell out, measured by staff member when patient readmitted to hospital
- Early unplanned return to hospital (yes/no) and reason, collected by staff member when patient readmitted to hospital

For PGs only:

- Total number of doses given (compare median number between groups)

For both methods:

- Discomfort during placement (NPRS, from 0 (no pain) to 10 (worst possible pain)), asked by staff member to the patient using a pictorial of the pain scale, measured in the first few minutes after placement
- Need for second method of cervical ripening (proportion of participants who need a second method, yes/no)
- Need for ARM (proportion of participants who need ARM, yes/no)
- Discomfort during ARM (NPRS, from 0 (no pain) to 10 (worst possible pain), asked by staff member to the patient using a pictorial of the pain scale, measured in the first few minutes after ARM

### Measured at time of birth

- Proportion of participants who had use of oxytocin infusion and at what cervical dilation it started (median score, from 0 to 10)
- Uterine hyperstimulation (yes/no)
- Proportion of participants who had use of epidural anaesthesia and at what cervical dilation it was placed
- Clinical diagnosis of chorioamnionitis (yes/no)
- Proportion of participants who had a clinically significant antepartum haemorrhage after start of IOL – cause, timing, and if associated with abnormal CTG
- Fetal blood sampling (lactate or pH) performed during labour (proportion of participants who had this done) and results (mean measurement)
- Mode of birth (proportion of participants who had spontaneous vaginal, assisted vaginal or caesarean section)
- Proportion of participants who had a vaginal birth within 24 h of start of IOL
- If caesarean section, reason and at what cervical dilation (mean score, from 0 to 10)
- Proportion of participants who had failed IOL (defined for the purposes of this study as caesarean section performed at < 4 cm)
- Uterine rupture (yes/no)
- Non-cephalic presentation (yes/no)
- Cord prolapse (yes/no)
- Stillbirth (yes/no)

### Measured post-partum before hospital discharge

- *Post-partum haemorrhage* (proportion of participants in each of three subgroups: 500–999 mL, 1000–1499 mL, 1500 mL or more), red blood cell transfusion (yes/no), transfer to theatre (yes/no)
- Clinical diagnosis of *post-partum endometritis* (yes/no)
- *Admission to intensive care* unit (primary diagnosis, outcome) (yes/no)

### Measured 4–8 weeks post-natal

- *Maternal satisfaction*, assessed by questionnaire

Fetal and neonatal:

### Measured at time of birth

- *Live birth* (yes/no);
- *Se*x (male/female)
- *5-min Apgar score* (median measurement, and proportion with score < 7)
- *Birthweight* (mean measurement in grams)
- *Birth injury* (severe bruising, nerve trauma, or fracture) (yes/no)
- *Arterial cord-gas results* (mean lactate or pH measurement, if pH then proportion < 7.0)

### Measured before hospital discharge

- *Admission to neonatal intensive care unit*/special care baby unit (yes/no)
- *Need for respiratory support* or mechanical ventilation (yes/no) and duration (time in hours will be calculated at the end of the study, based on times recorded by the staff member (from time of starting to time of stopping intervention), mean duration)
- *Length of stay* (time in hours will be calculated at the end of the study, based on times recorded by the staff member (from time of admission to time of discharge from the neonatal intensive care unit (NICU)), mean duration) (also proportion of admissions > 4 h)
- *Infection* (either culture proven or clinically suspected with supporting laboratory evidence such as raised white blood cell count or C-reactive protein) (yes/no)
- *Seizures* (yes/no)
- *Neonatal encephalopathy*, (yes/no, and if moderate or severe, and if due to hypoxic ischemic encephalopathy)
- *Early neonatal death* (yes/no)

Hospital:

- *Staff satisfaction*, assessed by questionnaire, measured annually at each participating hospital
- *Maternal length of stay*; (time in hours will be calculated at the end of the study, based on times recorded by the staff member (from time of admission to time of discharge from hospital), mean duration)
- *Neonatal length of stay*; (time in hours will be calculated at the end of the study, based on times recorded by the staff member (from time of admission to time of discharge from hospital), mean duration)
- *Pharmaceutical, equipment, and consumable costs*; health care utilisation costs; incremental cost-effectiveness ratio (ICER) for risk of caesarean section (see ‘Cost effectiveness analysis’ section)

**Fig. 1:**
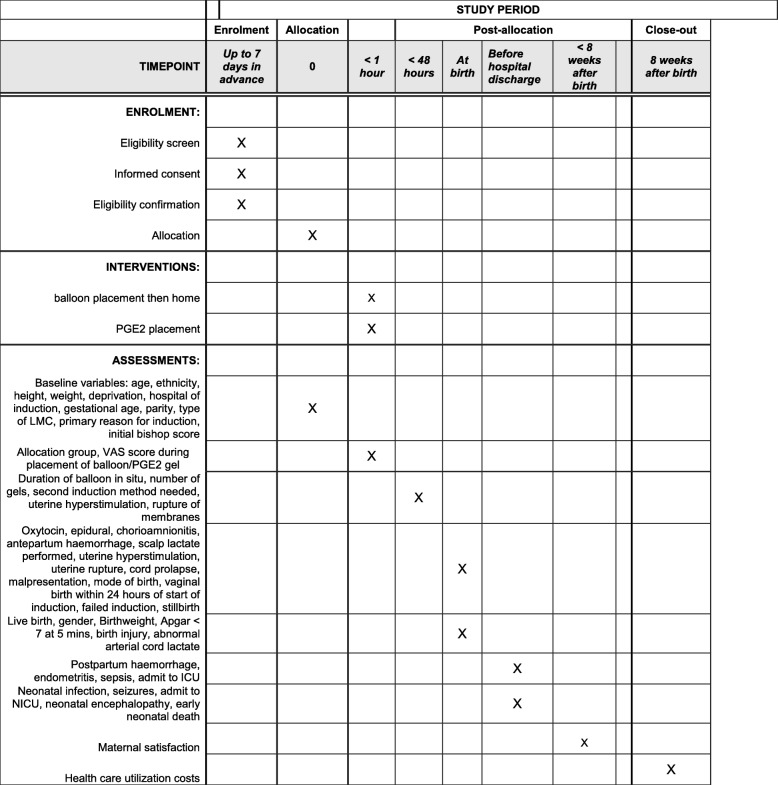
Schedule of enrolment, interventions, and assessments for the OBLIGE study

*Participant timeline*: see Fig. 1.

### Sample size calculation

At Auckland Hospital in 2015, the caesarean section rate in women who had undergone IOL (excluding women who had undergone previous caesarean section) was 24.8%, almost all of these women were induced using PGs [16]. In the small trial of outpatient vs inpatient balloon-catheter IOL, the risk of caesarean section rate decreased from 43 to 29% (a relative reduction of 32%) [11]. In order to detect a clinically meaningful change in risk of caesarean section rate from 24.8 to 18.8% (a relative risk reduction of 24%), with 80% power and a two-sided type 1 error of 0.05, we require a sample size of 743 women for each study group. Adding a continuity correction, the total sample size required is 1552 women.

### Recruitment

Potential participants are identified at the time of planning or booking an IOL, which can be between 1 day to 2 weeks prior to the IOL. Women are approached by a clinician or member of the research team to explain about the trial, encourage them to read the pamphlet or watch the video (both available at www.oblige.auckland.ac.nz), and answer questions. Inclusion and exclusion criteria are checked. Where possible, women’s interest in the trial is indicated in their paper-based or electronic medical records.

### Randomisation

On the day of IOL, after confirming eligibility, the clinician will obtain written consent from the participant. Participants will be randomised to one of two study groups, either the outpatient balloon-catheter group or the inpatient PG group, using a centralised, secure, online randomisation website. The randomisation schedule was prepared by the OBLIGE Trial Steering Committee (TSC). Randomisation is stratified by hospital and by parity (nulliparous or multiparous). Allocation is at a 1:1 ratio to outpatient balloon catheter: inpatient PG. The clinician, with support from the research team (if required), will perform the online randomisation, enrol the participant, and allocate the intervention to the participant.

### Blinding

Clinicians, who will be assessing the clinical outcomes, will not be blinded to treatment allocation; however, the outcomes are objective and most data are routinely collected during provision of clinical care. The researchers performing the data analysis, including the health economics analysis, will be blinded to group allocation.

### Data collection

Data will be collected using REDCap (https://www.project-redcap.org/), a secure web application for building and managing online surveys and databases. The data collection tool was developed and tested using an online, secure, central database specific to the OBLIGE trial. Researchers and clinicians involved in the trial receive their own user logon/password and undergo formal training to use the database.

The participant questionnaire (see Additional file 1) was developed for the purposes of this trial, and includes questions from the validated Childbirth Experience Questionnaire [17], questions recommended in the National Institute for Health and Care Excellence (NICE) guidelines on IOL [2], and questions from the patient satisfaction questionnaires from other recent maternity trials, provided on request to the PI (PINC – Dr M Beckmann; CLOSURE and GRoW – Prof J Dodd). The staff satisfaction questionnaire (see Additional file 2) was derived from the OPRA outpatient IOL trial (Prof D Turnbull).

As allocation to either balloon-catheter or PG intervention will occur within 1 h of randomisation, the intervention will be complete within about 48 h, and then all women will give birth within the next 48 h, we expect participant retention to be straightforward. Moreover, we do not anticipate many participants to deviate from their allocated method of induction.

One data collection challenge will be the measurement of the secondary outcome of maternal satisfaction 4 weeks after the birth. The use of an electronic post-partum questionnaire by return of email was successful in a recent local clinical trial of decision aids for women birthing after previous caesarean section [18], with an 82% return rate. Researchers will follow up by phone if participants do not return their electronic questionnaire. After two reminders, and if 8 weeks has passed, then the case will be closed.

### Data management

Data will be collected manually on pre-printed data collection forms by clinicians at the time of clinical care provision, then entered into the REDCap study database retrospectively by the local research team. A member of the central research team will check and clean the entered data with the forms, and raise queries if data are missing or unclear. Once queries are resolved the data are locked.

### Statistical analysis

Baseline demographic and clinical characteristics will be described for each study group. Analyses will follow the principle of intention-to-treat; participants will be analysed according to the assigned intervention group at randomisation. Multivariable models will control for potentially confounding variables and include hospital site and parity. Binary endpoints will be analysed using logistic regression to estimate odds ratios for the intervention. Continuous outcomes will be modelled using generalised linear models to estimate any changes in outcomes between the two interventions. A *p* value of 0.05 will be considered as statistically significant. We recognise that there are a number of secondary outcomes; as they are secondary outcomes we will report *p* values without correction, which would be overly conservative. Missing data will not be imputed.

### Cost-effectiveness analysis

Our approach will be to relate costs to outcomes for both arms of the study, allowing for the calculation of ICERs for the primary study outcome – caesarean section rate. In addition, a comparative cost analysis will be conducted to demonstrate the budget impact of wide-scale uptake of the intervention, given that length of stay and complication rates are higher for caesarean section. The approach taken will be to monitor costs as captured through utilisation for mothers and their babies from the point when IOL is initiated until discharge from hospital. Costs will be calculated using NZ Ministry of Health cost weights per event data which will allow for a summative total cost per delivery. Average costs will be compared between the study groups which will allow for the calculation of the net cost of the intervention. The net cost will be related to the primary outcomes of interest which will be then used to calculate ICERs for vaginal births and caesarean sections.

### Data monitoring

The Chair of the Data Safety and Monitoring Committee (DSMC) is a professor of obstetrics, and other members of the Committee are an academic obstetrician and an academic neonatologist (all associated with the University of Auckland). The DSMC defined the serious adverse events (SAEs) to be reported, prior to the start of the trial. The Chair of the TSC is the PI and the Committee includes a multidisciplinary team of researchers. The TSC and DSMC agreed that an interim analysis would be unnecessary.

### Harms

The database has alerts set for any occurrence of a SAE, which is reported to the PI, who informs the Chair of the DSMC. SAEs include: maternal death, maternal admission to intensive care, cord prolapse, stillbirth, neonatal sepsis, neonatal encephalopathy (moderate-severe), and early neonatal death. All severe adverse events will be reported in the publication of trial results.

### Audit

The PI performs an audit of trial conduct every 6 months and reports to the DSMC.

### Confidentiality

Participant’s personal information is collected on paper data forms. Data are entered into the secure online database. In Auckland, the paper forms are directly transferred to the locked office of the PI where they are kept in a locked cupboard. Forms from the other research sites are scanned and emailed to the Trial Manager for data checking, then kept securely in a locked cupboard or office of the local research team. The data will be de-identified prior to data analysis. Electronic data will be maintained in the database for 10 years as per ethics requirements in NZ, and paper forms from all sites will be placed in a secure university storage facility for the same duration.

### Dissemination policy

Results from this trial will be published in an appropriate peer-reviewed obstetrics journal. Authorship will be as per International Committee of Medical Journal Editors (ICMJE) guidelines. Researchers will also present results locally and nationally to other health care professionals. On the Consent Form (CF), participants are asked if they would like to receive a summary of the study results by email; this will be tracked in the database. Results will also be published on a public website www.oblige.auckland.ac.nz

## Discussion

This study has the potential to significantly change IOL practice in NZ and around the world. Paramount to all other outcomes from this study is that a simple intervention, such as a switch to the use of balloon catheters as the default method of IOL, may reduce the risk of caesarean section. Very few obstetric interventions studied in the last decade have resulted in reducing the caesarean section rate. A multi-faceted 2-year intervention in 32 Quebec hospitals managed to significantly reduce the caesarean section rate from 22.5 to 21.8% [19]. A 2-year quality improvement project in 56 California hospitals (the Toolkit Collaborative) found a significant reduction in caesarean section rate among standard nullipara, from 29 to 25% [20]. The OBLIGE trial is powered on the ability to detect a 24% relative reduction in risk of caesarean section, in a subset of women undergoing IOL. The ‘Choosing Wisely’ campaign has been put in place to facilitate wise decisions between service providers and patients about the most appropriate care for them, avoiding unnecessary interventions [21]. This study should provide the evidence to make such recommendations for IOL care provision.

Not only do most women want fewer interventions during childbirth (as long as they and their babies are safe), but women also want choice. If outpatient IOL with a balloon catheter is found to be both clinically effective and safe, then women who require an IOL can have some choice in how and where it will occur.

From a hospital service perspective, if outpatient balloon-catheter IOL is found to be cost-effective, then enabling women to access this method of cervical ripening has the potential to save significant resources in midwifery time, other resources and overall length of hospital stay. Hospitals have a great shortage of midwifery staff; thus, innovative solutions are needed to optimise the wise use of this resource. Moreover, the cost of a balloon catheter is much less than the cost of a single PG dose.

### Trial status

First participant recruited on 25 October 2017; anticipated recruitment completion December 2020; Study Protocol Version 9 dated 12 October 2018.

### Recruitment progress

Based on indications for IOL at Auckland Hospital [16], we estimated that 40% of women would be eligible for this trial. In keeping with previous trials, we estimated that 60% of eligible women would consent to participate. We additionally accounted for 5% drop out between randomisation and collection of primary outcome data.

We reviewed the number of IOLs at the hospital sites that expressed interest in participating in the trial, and the expected participants per annum ranged from 50 (Whakatane) to 870 (Auckland). Based on the assumption that all sites could start recruitment at the same time, we expected the overall study’s recruitment to take about 15 months (100 participants per month).

**Fig. 2:**
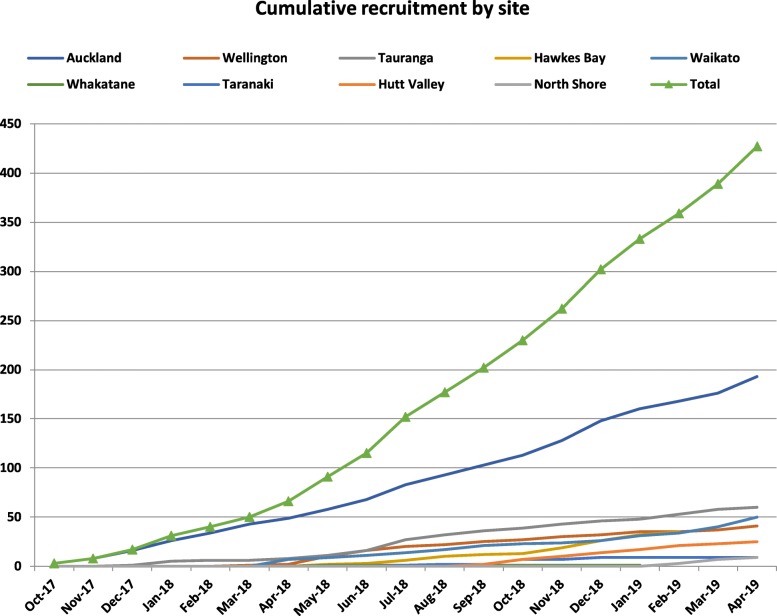
Monthly cumulative recruitment by site

The first site to start the trial was Auckland Hospital and the first participant was recruited in October 2017. During 2018, seven more hospitals signed research contracts to be involved in OBLIGE, and three in 2019. Monthly recruitment increased from five participants in November 2017 to 41 in May 2019 (see Fig. 2). The total recruitment to date is over 450 participants.

Recruitment has been slower than our estimates. On recent audit of three sites, eligibility rate was 40%, as expected. However, the acceptance rate was 25% which is much lower than expected. Based on revised practical estimates, we now anticipate completion of recruitment in December 2020.

## Supplementary information

**Additional file 1.** OBLIGE study satisfaction survey.

**Additional file 2.** Health professional survey.

## Data Availability

The final trial datasets generated and analysed during the current study will be accessible by the TSC, and to the DSMC on request. The corresponding author will also make the datasets available, on reasonable request, in order to contribute to an individual participant data (IPD) meta-analysis. De-identified trial data will be shared in accordance with the ICMJE data-sharing guidelines.

## Abbreviations

ANZCTR: Australian New Zealand Clinical Trials Registry
ARM: Artificial rupture of membranes
CF: Consent Form
CTG: Cardiotocography
DSMC: Data and Safety Monitoring Committee
HDEC: Health and Disability Ethics Committee
ICER: Incremental cost-effectiveness ratio
ICMJE: International Committee of Medical Journal Editors
IOL: Induction of labour
NPRS: Numeric pain rating scale
NZ: New Zealand
PGs: Prostaglandins
PI: Primary investigator
PIS: Patient Information Sheet
RANZCOG: Royal Australian and New Zealand College of Obstetrics and Gynaecology
RCT: Randomised controlled trial
RR: Risk ratio
SAE: Serious adverse event
TSC: Trial Steering Committee
