# Supplementary material for: Outpatient balloon catheter vs inpatient prostaglandin for induction of labour (OBLIGE): a randomised controlled trial
Source: Trials. 2020 Feb 17;21:190. doi: 10.1186/s13063-020-4061-5 (PMC7027046; doi:10.1186/s13063-020-4061-5)
Supplement: Supplementary file 1 — Additional file 1. OBLIGE study satisfaction survey. [file 13063_2020_4061_MOESM1_ESM.pdf]

OBLIGE Study Satisfaction Survey

Thank you for participating in the OBLIGE induction of labour study.

We would like to ask you some questions about your satisfaction with the care you received when you had your induction of labour. Information given will be kept strictly confidential.

Date of survey ______________________________

Thinking about how your induction was started this time (vaginal medicine and remaining in hospital OR balloon catheter and going home)

Would you choose to be induced again in your next pregnancy?
- ◯ Yes
- ◯ No
- ◯ Unsure

If you were to be induced in your next pregnancy, would you choose to start with:
- ◯ The same method I had this time
- ◯ A different method

	1 not at all	2	3	4	5	6	7	8	9	10 extremely satisfied
How satisfied were you overall with the start of your induction of labour?	◯	◯	◯	◯	◯	◯	◯	◯	◯	◯

Having the vaginal medicine or balloon catheter inserted was:
- ◯ Less painful than I expected
- ◯ More painful than I expected
- ◯ As painful as I expected

Overall, my induction of labour was:
- ◯ Less painful than I expected
- ◯ More painful than I expected
- ◯ As painful as I expected

At the following time points, please indicate your level of tiredness or fatigue.

	1 not at all tired	2	3	4	5	6	7	8	9	10 extremely tired
At the start of your induction of labour	◯	◯	◯	◯	◯	◯	◯	◯	◯	◯
At the time that you were transferred to labour and birthing suite	◯	◯	◯	◯	◯	◯	◯	◯	◯	◯

Approximately how many hours of sleep did you get on the night you had the vaginal medicine or balloon catheter inserted? ______________________________

Please rate whether you agree or disagree with each statement below.
(Please tick one option per line)

	Strongly disagree	Disagree	Neither agree or disagree	Agree	Strongly Agree
I was disappointed with how long it took for my labour to start after I had the vaginal medicine or balloon catheter inserted	○	○	○	○	○
I felt reassured having my induction started in this way	○	○	○	○	○
I felt well supported on the day I had the vaginal medicine or balloon catheter inserted	○	○	○	○	○
I had a good quality sleep on the night I had the vaginal medicine or balloon catheter inserted	○	○	○	○	○
It was convenient for me to start my labour induction in this way	○	○	○	○	○

Do you have any other comments about your experience?

____
