# Supplementary material for: Outpatient balloon catheter vs inpatient prostaglandin for induction of labour (OBLIGE): a randomised controlled trial
Source: Trials. 2020 Feb 17;21:190. doi: 10.1186/s13063-020-4061-5 (PMC7027046; doi:10.1186/s13063-020-4061-5)
Supplement: Supplementary file 2 — Additional file 2. Health professional survey. [file 13063_2020_4061_MOESM2_ESM.pdf]

Health Professional Survey

The OBLIGE research study on induction of labour has been up and running at Auckland DHB for over 6 months, and it is time to ask for your feedback about the study. It is for staff midwives and doctors at ADHB, and for Lead Maternity Carers; whether you have had several women in the study, or haven't even heard of it! By answering the following questions, we assume you consent to participate in this staff satisfaction survey. It should take only 5 minutes, and we appreciate any extra comments you wish to make. The results are anonymous. Thank you in advance for your time.

Dr Michelle Wise, Principal Investigator, OBLIGE Study
Senior Lecturer, Department of Obstetrics and Gynaecology, FMHS, University of Auckland

---

Thank you for contributing to the OBLIGE study. We would like to ask you some questions about the impact on you of having some women start their induction with a balloon as an outpatient. Your answers will be kept strictly confidential.

1. This section refers to the practice of induction of labour using a balloon

a. In your clinical role, how has the practice of balloon induction contributed to your satisfaction:

- ◯ improved my satisfaction a lot
- ◯ improved my satisfaction a little
- ◯ no difference
- ◯ decreased my satisfaction a little
- ◯ decreased my satisfaction a lot
- ◯ not applicable

---

b. How has the practice of balloon induction affected your stress at work:

- ◯ increased my stress a lot
- ◯ increased my stress a little
- ◯ no difference
- ◯ decreased my stress a little
- ◯ decreased my stress a lot
- ◯ not applicable

---

c. How has the practice of balloon induction affected your workload:

- ◯ increased my workload a lot
- ◯ increased my workload a little
- ◯ no difference
- ◯ decreased my workload a little
- ◯ decreased my workload a lot
- ◯ not applicable

---

d. In a typical work week, roughly how many balloon inductions would you be directly involved with: __________

---

e. Over the past 6 months, roughly how many balloon inductions were you directly involved with: __________

f. How would you rate your own experience with balloon induction overall:

- ◯ highly experienced
- ◯ somewhat experienced
- ◯ not at all experienced

2. This section refers to the practice of induction of labour as an outpatient

a. In your clinical role, how has the practice of outpatient induction contributed to your satisfaction at work:

- ◯ improved my satisfaction a lot
- ◯ improved my satisfaction a little
- ◯ no difference
- ◯ decreased my satisfaction a little
- ◯ decreased my satisfaction a lot
- ◯ not applicable

b. How has the practice of outpatient induction affected your stress at work:

- ◯ increased my stress a lot
- ◯ increased my stress a little
- ◯ no difference
- ◯ decreased my stress a little
- ◯ decreased my stress a lot
- ◯ not applicable

c. How has the practice of outpatient induction affected your workload:

- ◯ increased my workload a lot
- ◯ increased my workload a little
- ◯ no difference
- ◯ decreased my workload a little
- ◯ decreased my workload a lot
- ◯ not applicable

d. In a typical work week, roughly how many outpatient inductions would you be directly involved with:

____

e. Over the past 6 months, roughly how many outpatient inductions were you directly involved with:

____

f. How would you rate your own experience with outpatient induction overall:

- ◯ highly experienced
- ◯ somewhat experienced
- ◯ not at all experienced

3. To what extent do you agree with the following statement:

	Not at all satisfied	1	2	3	4	5	6	7	8	9	extremely satisfied
I am satisfied with my present job.	◯	◯	◯	◯	◯	◯	◯	◯	◯	◯	◯

4. Please provide comments about your experience so far with outpatient balloon induction as part of the OBLIGE research trial:

Things that I like about it

Things that I don't like about it

5. Please provide comments about recruitment for the OBLIGE research trial:

Things that can be barriers to recruiting women

Things that can help with recruiting women
